# Human Embryonic Stem Cell Responses to Ionizing Radiation Exposures: Current State of Knowledge and Future Challenges

**DOI:** 10.1155/2012/579104

**Published:** 2012-08-16

**Authors:** Mykyta V. Sokolov, Ronald D. Neumann

**Affiliations:** Nuclear Medicine Division, Radiology and Imaging Sciences Department, Clinical Center, National Institutes of Health (NIH), 9000 Rockville Pike, bldg 10, room 4D49, Bethesda, MD 20892, USA

## Abstract

Human embryonic stem cells, which are derived from the inner cell mass of the blastocyst, have become an object of intense study over the last decade. They possess two unique properties that distinguish them from many other cell types: (i) the ability to self-renew indefinitely in culture under permissive conditions, and (ii) the pluripotency, defined as the capability of giving rise to all cell types of embryonic lineage under the guidance of the appropriate developmental cues. The focus of many recent efforts has been on the elucidating the signaling pathways and molecular networks operating in human embryonic stem cells. These cells hold great promise in cell-based regenerative therapies, disease modeling, drug screening and testing, assessing genotoxic and mutagenic risks associated with exposures to a variety of environmental factors, and so forth. Ionizing radiation is ubiquitous in nature, and it is widely used in diagnostic and therapeutic procedures in medicine. In this paper, our goal is to summarize the recent progress in understanding how human embryonic stem cells respond to ionizing radiation exposures, using novel methodologies based on “omics” approaches, and to provide a critical discussion of what remains unknown; thus proposing a roadmap for the future research in this area.

## 1. Introduction

Human pluripotent cell lines have been derived from the inner cell mass of the preimplantation embryos (embryonic stem cell lines, hESC) [1] and from fetal germ cells (embryonic germ cell lines, hEGC) [2] demonstrating a stable developmental potential to form advanced derivatives of all three embryonic germ layers for prolonged periods of maintenance in the undifferentiated state in culture. Studies of hESC lines have numerous implications for human developmental biology, drug discovery, drug testing, and cell-based regenerative medicine. Since their initial isolation in culture in 1998 by Thomson, many aspects of hESC biology have been already illuminated. At the same time, wide gaps in our knowledge about the basic hESC biology still remain to be filled. One of the less-studied areas pertaining to hESC biology is the response of these pluripotent cells to genotoxic stress exposures. This has only recently begun to attract due interest from the stem cell researchers even though its importance is paramount. The maintenance of genome fidelity over the course of the earliest stages of human development is crucial for the faithful reproduction and, hence, for the survival of the human as a biological species. Therefore, the mechanisms that serve to protect the developing embryos at one of the most vulnerable stages of human development from the genotoxic effects of endogenous and exogenous agents such as ionizing radiation (IR) and oxidative stress must be examined and fully understood before the full promise held by hESCs can be realized in applied medicine.

The objective of this paper is to describe the current state of knowledge of hESC response to IR exposures and to discuss possible future directions in research. Particular emphasis will be given to summarizing recent experimental studies that focus on the survival of irradiated hESCs, signaling networks perturbed by IR exposures, and hESC potential for multilineage differentiation *in vitro* and *in vivo* following irradiation. We will also outline key scientific questions that remain to be addressed in a future studies in order to foster the translation of basic discoveries pertaining to hESC into medicine.

IR represents a type of electromagnetic radiation produced naturally by cosmic rays, radioactive isotopes present in an Earth' crust, as a result of human activities associated with diagnostic and therapeutic procedures in clinic and medicine (X-rays, computed tomography (CT)-scans, fluoroscopy, positron emission tomography (PET), radiotherapy, etc.), as well as nuclear power plant environmental catastrophes, such as those occurred in Chernobyl and Fukushima Daiichi. In addition, concerns are put forth regarding the probability of so-called “dirty bomb” radiological attacks by terrorists, which would also result in emission of IR. IR exposures are known to elicit a complex spectrum of biological responses in humans, including, but not limited to, mutagenesis, carcinogenesis, teratogenesis, and cell killing. Some of these effects are probabilistic and others are deterministic in nature [3–5]. Moreover, some biological effects of IR could manifest rather early after IR exposures; and, on the opposite, some of these effects may take decades for their full development.

The earliest stages of human development are considered by many to be among the most sensitive and vulnerable to damaging effects of IR. However, current consensus is that exposure to radiation of less than 5 cSv during pregnancy is not associated with an elevated risk of malformation [6, 7]. But this assumption is based on very limited human data and/or on animal models, and thus may not accurately reflect the human embryonic response to IR exposures. Hence, the potential for damage caused by IR of different types and levels of exposures to the early embryo is still largely unknown, leading to uncertainties in corresponding risk estimates.

With the growing number of experimental studies utilizing induced pluripotent stem cell-(iPSc) and hESC-based approaches to provide treatment of different types of diseases, research into hESC responses to IR exposures becomes a “hot” topic. The necessity to track stem cell- and/or committed progenitor's fates in cell-based regenerative therapies in clinical settings may require the use of imaging tests producing IR exposures, such as CT-scans, and/or PET- or single-photon emission-computed tomography (SPECT). For SPECT reporter probes are being developed to monitor stem cell transplantation; in these cases, irradiation of stem cells is inevitable [8, 9]. Therefore, studies of hESC responses to IR exposures could yield novel insights into both basic and applied biology.

## 2. Human Embryonic Stem Cell Fate after Exposures to Ionizing Radiation

The cellular radioresponses of many different types of differentiated cells representing many of the tissues constituting humans were examined to date, including fibroblasts, keratinocytes, and muscle cells [10–15]. The fate of these irradiated cells varies widely depending on a dose, dose-rate, linear energy transfer (LET) of radiation, microenvironment, and other factors; different types of cell death, cell cycle arrest, senescence, quiescence, genomic, and epigenomic instabilities may result as a consequence of IR exposures. Until recently, however, very little was known how hESCs respond to IR.

**Figure 1:**
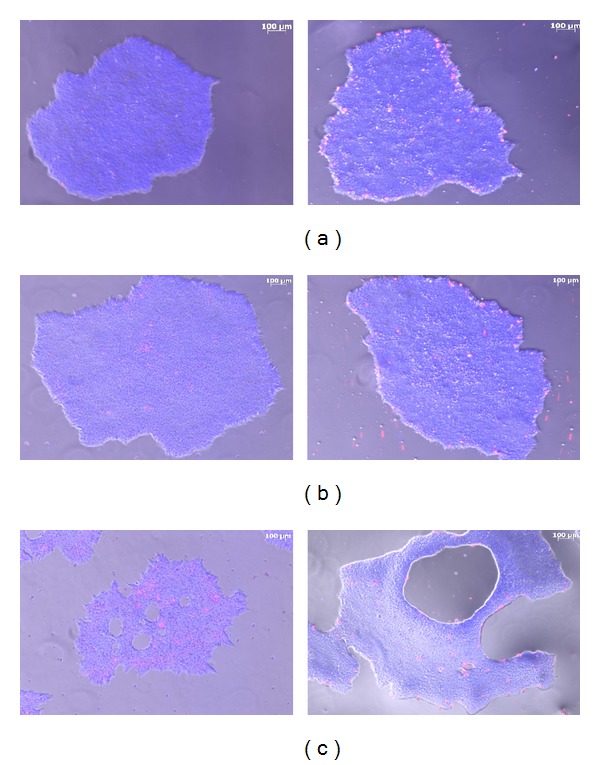
Induction of apoptosis in hESC cultures after IR exposures. Shown are merged images of H9 hESC colonies (blue—Hoechst 33342, red—propidium iodide). (a) sham-irradiated hESC cultures (0 Gy), (b) 0.2 Gy exposure, (c) 1 Gy IR exposure. Left panel—6 hr post-IR, right panel—41 hr post-IR.

An accumulating body of evidence suggests that these pluripotent cells readily undergo apoptosis beyond the low-dose IR exposures. Indeed, we did not observe an increase in apoptotic cell incidence after 0.05 Gy and 0.2 Gy of X-ray exposures (Figure 1(b)). At a higher dose of 1 Gy, a robust apoptotic response was evident (Figure 1(c)). Other groups reported massive cell death coinciding with the development of holes and patchy regions in hESC colonies at 48 hours after 4 Gy of IR exposures [16]; the formation of holes was also shown in colonies 6 hours following 5 Gy irradiation [17]. Exposures of H1 line of hESCs to 5 Gy of gammaradiation decreased cell viability by approximately 65% at 7 h after treatment [18].

Apparently, there is a trend toward increasing apoptosis and resultant cell killing at the higher radiation doses (2 and 4 Gy) compared with low dose (0.4 Gy) or sham-irradiated hESCs. It was found that the majority (>70%) of hESCs undergo cell death after 4 Gy irradiation, although a subfraction of these cells was still viable at 48 hours [16]. However, we and others showed that the surviving hESCs continued to express common pluripotency markers, such as TRA-1-81, SSEA4, and TRA-1-60, and embryonic transcription factors, such as Oct4, Sox2, and Nanog, which are key regulators of pluripotency and self-renewal [16, 17, 19].

Interestingly, other types of radiation, such as UV result in a similar hESC fate. Five hours after UV exposures (20 J/m^2^) of both H1 and H9 hESCs, the cells began to undergo apoptosis; after 12 h, 67% of the UV-irradiated cell were dying by apoptosis or necrosis; after 40 h all of the UV-irradiated cells were found to be dead [20]. It was shown that p53 is rapidly induced by UV; p53 promoted apoptosis by activating the mitochondrial pathway through caspase 9 (nearly 3-fold induction) [20].

Very recently, the robustness of apoptosis induction following genotoxic exposures in hESCs has been postulated to depend on constitutively active Bax sequestered at Golgi. The active Bax undergoes p53-dependent translocation to mitochondria to initiate the release of proapoptotic factors and trigger the suicide program [21]. Therefore, these differences in detail underpinning molecular mechanisms of apoptosis operating in hESCs and differentiated human cells may explain the propensity of hESCs to undergo programmed cell death in response to genotoxic stresses including IR exposures.

## 3. Effects of Ionizing Radiation Exposures on the Cell Cycle of Human Embryonic Stem Cells

One of the most studied consequences of IR exposures is alteration of the cell cycle of exposed human cells. Recently, mechanistic insights into the basic characteristics of hESC division have begun to accumulate. Early studies show that hESCs (H1 and H9 lines) possess a very short cell cycle (15-16 h) compared with human differentiated somatic cells, such as normal diploid IMR-90 fibroblasts [22].

The hESC cell cycle maintains the four known cell cycle stages, G1, S, G2, and M, but the duration of G1 is substantially attenuated (only about 2.5–3 h). Interestingly, about 65% of asynchronously growing hESCs reside in S phase at any given moment of time [22]. S phase lasts approximately 8 h, G2 was shown to last approximately 4 h, and M phase, about 1 h, which is in a good agreement with earlier data obtained for differentiated human cells. Detailed molecular analyses demonstrated that hESCs and differentiated cells express similar cell cycle markers. However, higher levels of expression of G1 phase-related *CDK4* and *CCND2* genes were found in hESC [22]. Thus, a molecular signaling network operative in hESCs may expedite the cellular progress into S phase to start DNA replication and histone protein biosynthesis in order to form new chromatin. Importantly, hESCs differ from somatic cells in the expression of the E2F family members and RB family pocket proteins, such as p105 (RB1), p107 (RBL1), and p130 (RBL2/RB2) governing expression of genes encoding enzymes for nucleotide metabolism and DNA synthesis [23]. Following IR exposures, histone gene expression reduces; for example, IR causes accumulation of unprocessed histone H4 precursor RNAs [23].

In more detailed subsequent studies addressing the molecular mechanisms of histone gene expression in hESCs, it was reported that HiNF-P/p220 gene regulatory pathway responsible for histone H4 expression is fully functional in hESCs. It supports expression of DNA replication-linked histone genes and chromatin assembly to foster hESC self-renewal [24]. The temporal characteristics of the formation of histone locus bodies imply that the G1 phase of the cell cycle in hESCs is shortened in part by contraction of late G1 [25]. It was concluded that cyclin D2 and p220 (NPAT) are key cell cycle regulators underlying competency for self-renewal in hESCs [26].

Notably, E2F4, E2F5, and p130 (RBL2/RB2) were shown to be the major E2F and pocket protein transcripts in unstressed hESCs. Whereas, the expression levels of E2F5, E2F6, and p105 (RB1) transcripts were robustly elevated during cell cycle arrest in hESCs after IR [23]. Such genotoxic stress response in hESCs may ultimately alter the E2F/RB-related combinations underpinning E2F-dependent genes and (i) inhibit histone-specific transcription factors, (ii) delay processing of histone gene transcripts, and (iii) destabilize histone mRNAs [23].

 Therefore, hESCs demonstrate unique G1 cell cycle parameters and may use distinct cell cycle machinery that by passes E2F/pRB-dependent growth control to maintain self-renewal and pluripotency as compared with fully differentiated human somatic cells [27–30]. This, in turn, underlies IR-specific responses of hESCs. The integral part of such a response is a DNA damage response (DDR) coordinating alterations in cell metabolism, DNA repair, and cell cycle arrest following IR exposures.

One of the key molecular events responsible for activating DDR in response to DNA double-strand breaks (DSBs) is the induction of the ataxia telangiectasia mutated (ATM) signaling pathway. It was reported that in hESCs ATM kinase is phosphorylated and localized to the sites of DNA DSBs within 15 minutes of IR exposures [17]. Phosphorylation of ATM at serine 1981 was detected in hESCs one hour following exposure to two grays of *γ*-radiation. ATM activation was steady until four hours following IR exposures, at which time the levels begin to decline, but remained above control ATM levels in sham-exposed hESCs for at least 24 hours [17]. Activation of ATM triggered the phosphorylation of its downstream targets, such as p53, Chk2, and Nbs1. H2AX has been shown to be a target for phosphorylation by ATM in human somatic cells [31], although the relative role of ATM in H2AX phosphorylation in IR-exposed hESCs is not clear; some studies suggest the role for ATR in this process [32]. Phosphorylation of Chk2 at threonine 68 peaked at one hour following IR and eventually declined; so that only a minor fraction was still phosphorylated at six hours after IR. Nbs1 phosphorylation at serine 343 followed a similar with pChk2 time course [17]. The number of *γ*-H2AX ionizing radiation-induced foci (IRIF) increased immediately following IR and returned close to levels seen in unstressed hESCs within 24 hours.

We observed the same trend with 53bp1 IRIF as well [33]. Within one hour of irradiation, phosphorylation of p53 on serine 15 and serine 20 was detected, reached maximal levels by two hours, and declined afterwards; but it still remains elevated above control levels for 24 hours [17]. Importantly, IR exposures resulted in a temporary cell cycle arrest at the G(2)/M phase, but not G(1)/S phase, after 2 Gy dose of gamma-radiation [17]. We also reported that hESCs lack G(1)/S phase arrest after IR exposures [33]. Notably, hESCs overcome cell cycle arrest approximately 16 hours after IR, having a 4-fold higher incidence of aberrant mitotic figures representing mitotic spindle defects compared with sham-exposed hESC cultures. At 48 hours after IR, the cell cycle distribution closely resembled that of nonirradiated cells. ATM was found to play an essential role in establishing G(2)/M arrest since ATM inhibition resulted in abrogation of G(2)/M arrest, manifested by a decrease in a number of arrested cells just 2 hours after IR exposures [17].

These reports and our own results indicate that hESCs activate DDR [17, 29, 33], resulting in an ATM-dependent G(2)/M arrest. However, hESCs reenter the cell cycle with many cells bearing mitotic spindle defects [17]. Importantly, some data suggest that mitotic spindle checkpoint functions in hESCs, but is uncoupled from apoptosis [34]. These abnormal hESCs could potentially be eliminated during further checkpoint, or could undergo apoptosis when bearing these mitotic spindle defects. Interestingly, UVC exposures of hESCs in G1 phase led to cell cycle arrest before DNA synthesis and to a decreased CDK2 activity [35]. However, p21, the main constituent of G(1)/S checkpoint induction in fully differentiated human cells, was found not to be responsible for the cell cycle progression pause in UVC-exposed hESCs. In marked contrast, upon CDK2 downregulation with siRNA p21 was shown to be increased as a late event (day 4) associated with DDR and G(1)/S checkpoint activation [36]. Therefore, DDR appears to be highly context-dependent in hESCs. Importantly, some data suggest that p21 was robustly activated by genotoxic stresses such as IR exposures at the transcript level in hESCs (about 15-fold 2 hr after 5 Gy of irradiation) [18]. Intriguingly, amount of p21 protein in hESCs was barely elevated after IR [18]; it implies that p21 gene is robustly expressed but the protein is only weakly translated in IR-exposed hESCs. It is possible that the levels of p21 in irradiated hESCs are not enough to inhibit cyclin-dependent kinases, such as CDK2, to elicit G(1)/S checkpoint. In marked contrast, hESCs treatment with agents promoting differentiation, such as nutlin and sodium butyrate, rapidly induces p21 both at transcript and protein levels [37]. Hence, by escaping G(1)/S checkpoint following DNA damage, hESCs might be minimizing the risk of spontaneous differentiation. Another interesting aspect of IR exposures of hESCs could be the cell cycle-dependent induction of apoptosis, which may predominantly occur in S phase in these cells [21]. The lack of G(1)/S arrest in irradiated hESCs may promote the apoptotic clearance of cells bearing DNA damage inflicted during G1 phase of cell cycle, since the error-free DNA repair by homologous recombination is not operative at this point. Therefore, it seems that pluripotent hESCs are capable of activating the G(1)/S checkpoint upon DDR induction, at least under specific genotoxic stress exposures, although the molecular mechanisms of such arrest may differ between hESCs and adult somatic human cells.

## 4. DNA Repair in Human Embryonic Stem Cells after Exposures to Ionizing Radiation

DNA repair mechanisms are known to be responsible for preserving genomic integrity in all human cell types. The major DNA repair pathways operating in human cells include base excision repair (BER) [38, 39], nucleotide excision repair (NER) [40, 41], mismatch repair [42], homologous recombination repair (HRR) [43, 44], and non-homologous end-joining (NHEJ) [45, 46]. BER is involved in correction of small DNA alterations, such as oxidized bases, or alkylating agent damage. In contrast, NER is shown to remove mainly bulky lesions, such as cyclobutane pyrimidine dimers by excision of oligodeoxyribonucleotides. HRR, and NHEJ are different pathways that repair DNA DSBs [47]. Error-free HRR requires a homologous DNA template; in marked contrast, a homology is not a prerequisite for NHEJ, which makes this pathway more error-prone. Mismatch repair is known to guard the genome against mismatched bases or single-strand loops. Although some repair pathways are inherently error-prone, for all of these mechanisms, low-fidelity DNA repair can result in genomic alterations such as mutations and/or translocations, thereby increasing the chances for cell transformation, and other deleterious consequences such as accelerated onset of age-related diseases at the organism level [48]. Hence, the functional characterization of DNA repair pathways in hESCs is a necessary prerequisite for prospective cell-based regenerative therapies, as well as for understanding how pluripotent human stem cells protect their genomes from such damage.

Recently, a number of publications focused on examining DNA repair pathways in hESCs highlighted the overall increased efficacy of removing the molecular damage from genomic blueprint in these pluripotent human stem cells compared to fully differentiated human cells [32, 49–52]. DNA DSB repair at a targeted break site is highly precise in hESCs, compared to somatic human cells [51]. Directed differentiation of hESCs into astrocytes reduces both the efficiency and fidelity of repair. Interestingly, it was demonstrated that the frequency of HRR event at a single DNA DSB differs up to 20-fold between otherwise isogenic hESCs based on the site of the DSB within the genome [51]. Thus, DNA DSB repair outcomes can differ based on both the location of the site of damage within the genome and/or the stage of cell differentiation.

Studies done by another group showed that DNA DSB repair in hESCs is more complex than repair both in neural progenitors (NPs) and astrocytes. The resolution of gamma-H2AX foci that served as a surrogate marker for DNA DSBs occurred at a slower rate in hESCs compared to NPs and astrocytes [32]. In addition, the dynamics of RAD51 foci, reflective of active HRR, indicates that hESCs as well as NPs have high capacity for HRR. Interestingly, ATM kinase was shown not to be critical for foci formation in hESCs, suggesting that the DNA damage response (DDR) is different in hESCs and differentiated human somatic cells [32]. The ability of hESCs to form IRIF was abrogated with caffeine and siRNAs targeted against ATR, suggesting that hESCs rely more on ATR, rather than ATM for executing DNA DSB repair. This relationship reverted to the opposite pattern as cells differentiated. Therefore, hESCs were found to have efficient DNA DSB repair, that is, largely ATR-dependent HRR [32].

In addition to predominant HRR operating in hESC to perform DNA DSB repair, hESCs, as other types of human cells, also use NHEJ. But, major differences were revealed between isogenic hESCs and more committed cells undergoing directed differentiation. Indeed, NHEJ kinetics was shown to be several-fold slower in hESCs and NPs than in astrocytes derived from hESCs [50]. ATM and DNA-PKcs inhibitors were ineffective or only partially effective, respectively, at inhibiting NHEJ in hESCs. DNA-PKcs knock-down in hESCs did not result in any major alterations in NHEJ in hESCs [50]. Poly(ADP-ribose) polymerase (PARP) was dispensable for NHEJ suggesting that DNA repair is largely independent of backup NHEJ. Importantly, as hESCs progressively differentiated a proportional decrease in the accuracy of NHEJ occurred [50]. Therefore, NHEJ in hESCs is largely independent of ATM, DNA-PKcs, and PARP; interestingly, NHEJ appears to be dependent on XRCC4 with repair fidelity several-fold greater in hESCs than in human differentiated cells such as astrocytes.

Different types of DNA damage induced by H_2_O_2_, UV-C, IR exposures, or psoralen undergo more efficient repair in hESC (lines BG01 and I6) compared with human primary fibroblasts (lines WI-38, HS27) and, with the exception of UV-C damage, HeLa cells [49]. DNA microarray gene expression studies showed that transcript levels of several DNA repair genes are increased in hESCs compared with their differentiated forms such as embryoid bodies [49].

Importantly, DNA repair capacities of hESCs (H9, BG01, and BG01V lines) and induced pluripotent cell lines were found to be more heterogeneous than those of differentiated cell lines (IMR-90, HF01, HF02, and HF51) examined in a study by Luo et al. [52]. Although pluripotent cells were shown to possess high DNA repair capacities for NER, low level UV exposures induced an apoptotic response in hESCs. On the contrary, under these same conditions somatic differentiated cells failed to mount a similar response, proving to be less vulnerable to genotoxic stresses in terms of cell survival [52]. Interestingly, human pluripotent cells including hESCs were found to undergo a similar apoptotic response to alkylating agent DNA damage. Notably, human pluripotent cells that survived UV exposures exhibited less DNA damage compared with differentiated cells that received the same UV flux. Together, these data suggest that genomic maintenance pathways are in general enhanced in hESCs, relative to differentiated human cells. In addition, these results underscore the importance for investigators to evaluate DNA repair capacities in hESCs, and to characterize their genomic stability, prior to any possible application in clinical trials.

## 5. Transcriptional Responses of Human Embryonic Stem Cells to Ionizing Radiation

One of the key consequences of exposures of human cells to genotoxic agents, including IR, is the change in the expression level of multiple genes [13, 53, 54]. While the mechanisms underpinning radiation-induced gene expression alterations in fully differentiated somatic human cells have been studied extensively, molecular signaling events and pathways involved in global transcriptional responses of pluripotent hESCs until recently remain unexplored. At present, only two published papers addressed this lack of knowledge.

DNA microarray technique was used to analyze the global gene expression changes in H9 cell line of hESCs 24 hours after 0.4 Gy, 2 Gy, and 4 Gy of gamma-radiation [16]. The Gene Ontology analysis at each radiation dose identified biological processes and pathways that are involved in cell death, cell cycling, p53 signaling, cancer, embryonic and organ development, and others. It was shown that the expression of a set of core transcription factors defining pluripotency in hESCs is not altered by radiation at any dose up to 4 Gy [16]. Interestingly, obvious coclustering of the control and relatively low dose IR cultures of hESCs was observed (0 and 0.4 Gy), which was distinct from the co-clustering of the higher doses hESCs samples (2 and 4 Gy) [16].

Relatively low dose (0.4 Gy) of gamma-radiation was shown to affect cellular functions such as cell death, cancer, and p53 signaling pathways; however, some important p53 downstream target genes such as *CDKN1A* and *HDM2 *were identified as being nonresponsive to 0.4 Gy. Apparently, these findings contradict the findings made previously with fully differentiated human cells [55], and underscores the differences in radioresponses between hESCs and adult somatic cells. Relatively low dose 0.4 Gy was found not to reduce hESCs proliferation to an extent comparable to higher dose exposures. Since *CDKN1A* is widely regarded as an important negative regulator of cell cycle progression arresting cells at G(1)/S [56], the lack of upregulation of *CDKN1A* with 0.4 Gy IR exposures could potentially explain this observation, at least partly.

Compared to 0.4 Gy irradiation, 2 Gy irradiation affects canonical TFG-*β* and Wnt/*β*-catenin signaling, including *WNT10A* (up 2.1-fold), *WNT9A* (up 1.8-fold), and *TGFBR2* (up 1.4-fold). The differential modulation of Wnt gene expression following IR exposures could potentially result in profound alterations in hESC biology, since these genes are known to be implicated in key signaling pathways in hESCs. The higher dose of radiation (2 Gy) also induced *CDKN1A* overexpression by 2.3-fold, but not *HDM2*. Notably, many genes involved in general metabolism functions such as amino acid metabolism, molecular transport, such as *SLC6A13* (up 2-fold) and *SLC25A13* (down 2.2-fold), and cell morphology, in addition to cancer and cell death, were shown to be significantly deregulated in hESCs by 2 Gy of radiation.

Finally, in the 2 Gy versus 4 Gy group, the overall gene changes were not observed to be different to a large extent, but a small group of genes related to organ and tissue development was found to have an altered expression, such as *TNFSF11* (up 1.6-fold), *OTX1* (down 1.6-fold), *B4GALT1* (down 1.4-fold), and *MEF2C* (up 1.9-fold). After 4 Gy irradiation, biological processes/pathways such as p53 signaling, VDR/RXR activation, aryl hydrocarbon signaling, and functions such as cancer, cell death, cell cycle, proliferation, and embryonic development were identified as being significantly affected in hESCs. Several p53 associated and regulated genes such as *TP53INP1 *(up 2.6-fold), *CDKN1A* (up 2-fold), and *HDM2* (up 1.7-fold), as well as several tumor necrosis factor (TNF) receptor superfamily members, were shown to be induced after IR. A small group of genes implicated in development processes also exhibited differential expression, including *RUNX1* (up 1.5-fold), *HES1* (down 1.8-fold), and *PBX1* (down 1.8-fold). Perhaps some minute changes in the development and differentiation processes occurred with 4 Gy irradiation in hESCs, but these alterations were not strong enough to cause loss of pluripotency, as was evidenced by (i) maintenance of expression level of the core transcriptional factors, and (ii) successful formation of teratomas from 4 Gy-irradiated hESCs. Clearly, gene expression changes in hESCs are dose-dependent at 24 hr after IR, but the dynamics of transcriptional responses of hESCs to IR exposures was not clarified, since only one timepoint after IR was analyzed.

To fill this gap in knowledge, we aimed to study changes in the human genome-wide transcriptome of H9 hESC line following exposures to 1 Gy of gamma-radiation at 2 h and 16 h after irradiation, thus elucidating the “early” and “late” hESC radioresponses, respectively [33]. We found that the changes in gene expression in hESCs after IR exposures are substantially different from those observed in somatic human cell lines. Gene expression patterns at 2 h post-IR were characterized bearing almost an exclusively p53-dependent, predominantly proapoptotic, signature with a total of only 30 upregulated genes. Many of induced “early”-response genes were already shown to participate in radioresponse in human adult differentiated cells, such as fibroblasts and peripheral blood cells [13, 54, 57]. Indeed, *BTG2* (up 6.6-fold), *CDKN1A* (up 5.8-fold), *SESN1* (up 3.5-fold), *IER5* (up 3-fold), and *GADD45A* (up 2.8-fold) are found among the best studied and thoroughly characterized markers of IR exposures of human cells; and the induction of these genes is usually considered to be associated with temporal cell cycle arrest. For example, *GADD45A* is implicated in G(2)/M arrest and *PLK2 *(up 4.3-fold) and *PLK3* (up 4.3-fold) are involved in G(2)/M transition of the cell cycle.

Importantly, several of “early” response genes in irradiated hESCs are known to participate in apoptosis, such as GDF15 (up 5.2-fold), *BBC3 *(up 3.5-fold), *HTATIP2* (up 3.5-fold), *CARD8* (up 2.9-fold), *FAS *(up 2.8-fold), and *TP53INP1* (up 2.7-fold) [33]. A few up-regulated gene products in hESCs at 2 h after 1 Gy IR exposures belong to zinc finger protein superfamily, such as *ZNF79* (up 6.9-fold), *ZNF761* (up 3-fold), *ZSCAN20* (up 2.5-fold), and *ZNF135* (up 2.5-fold); they may function as transcription factors.

In marked contrast, the gene expression patterns at 16 h after IR showed 354 differentially expressed genes in hESCs, with many genes involved in prosurvival pathways, such as increased expression of metallothioneins, ubiquitin cycle, and general metabolism signaling. Interestingly, all of them were found to be up-regulated, with the magnitude of expression varying in range from about 1.5-fold till 25-fold over time-matched mock-treated cell cultures. The open hESC chromatin structure, enriched in noncompact euchromatin, may allow easy access for transcription factors and the transcriptional machinery, and may explain observed lack of downregulation in gene expression after IR exposures. Irradiated hESCs robustly overexpressed many genes belonging to metallothionein gene superfamily, such as *MT1M* (up 5.1-fold), *MT1L* (up 3.1-fold), *MT1H*, and *MT1G*, which is also observed in many types of differentiated human cells after IR [13, 54, 57]. The metallothioneins are known to be implicated in the protection of cell populations from the oxidative stress. A few members of histone gene superfamily were shown to be strongly modulated at 16 hr timepoint after IR exposures, such as *HIST1H4I* (up 22.9-fold), and HIST1H4E. However, the relevance of overexpression of these specific histone species in irradiated H9 cells remains to be studied. Many transcription factors were identified as being radioresponsive at 16 hr after IR; among them *ZNF302* (up 6.6-fold), *SP5* (up 5-fold), *ZNF33A* (up 3.8-fold), *ZNF697* (up 3.4-fold), *ZFYVE16* (up 3-fold), and others.

Our DNA microarray analysis of IR-exposed H9 hESCs revealed that the gene expression signatures characterizing “early” (2 h) and “late” (16 h) radioresponse to 1 Gy are fundamentally distinct. We found that only six genes were overexpressed at both timepoints examined; they were *CDKN1A, BTG2, GDF15, SESN1, PLK3*, and *ANKRA2*. Sustained expression of these genes in irradiated hESC may constitute the specific “gene expression signature” which could potentially serve as a marker of IR exposure of hESCs, although further experiments including dose-response and additional time-course studies are definitely warranted for a proper validation of this finding.

## 6. Epigenetic Regulation of Responses of Human Embryonic Stem Cells to Ionizing Radiation Exposures

Epigenetics embraces studies of heritable changes in gene expression or alterations in phenotype caused by mechanisms other than changes in the nucleotide sequences of the genome. Known examples of such investigative efforts is research into DNA methylations and histone modifications, both of which regulate gene expression without altering the underlying DNA sequence. Almost nothing is known how hESCs responses to IR are affected by epigenetic landscape of these cells which is quite unique in terms of chromatin “openness” and much less prevalence of heterochromatin compared to differentiated cells [58–60]. It remains to be seen in the future if patterns of DNA methylations are altered in irradiated hESC compared to sham-exposed cultures; and, as a result, how DNA methylation alterations would influence gene expression changes. It is known that DNA methylation patterns in hESCs are distinct from those in fully differentiated somatic human cells in several respects. First, so-called histone bivalent marks are associated with promoters of many developmentally regulated genes. Second, a much higher abundance of non-CG DNA methylation observed in hESCs [61–64].

Another important mode of epigenetic control of hESC responses to IR exposures is mediated by changes in the global microRNAome in these cells. MicroRNAs (miRNA) comprise a group of short ribonucleic acid molecules implicated in regulation of key biological processes and functions at the posttranscriptional level. Previous research indicates that many miRNA species are expressed predominantly in pluripotent hESCs, and as such represent hESC-specific miRNAome signature [65, 66]. Recent evidence using UV-radiation of hESCs showed that expression of p21 protein is directly regulated by the miRNA pathways under both regular cell culture conditions and following irradiation. Tens of miRNA species were upregulated after UV-exposures, including hESC-specific miRNAs such as those of the miR-302 cluster and miR-371-372 family. Members of miR-302 family, enriched in hESCs, such as miR-302a, miR-302b, miR-302c, and miR-302d, have been shown to be directly involved in regulation of p21 expression in hESCs, demonstrating a novel function for miR-302 s in hESCs [67]. Importantly, miR-372 negatively regulates the expression of p21 in unstressed hESC which is necessary for unperturbed hESC division to proceed [68].

The role of miRNAs in IR-induced responses in hESCs has only recently begun to be addressed. By using system biology approaches, we showed for the first time, that the miRNAome undergoes global alterations in hESCs of two distinct lines (H1 and H9) after IR. Genome-wide analysis of expression levels of 1,090 miRNA species in irradiated hESCs showed statistically significant changes in 54 genes following 1 Gy of X-ray exposures [69]. We found that global miRNAome alterations are highly temporally and cell line-dependent in hESCs. Research into the dynamics of transcriptional response of miRNAome to IR revealed that the 16 hr after IR radiation response of hESCs is much more robust compared to 2 hr-response signature in terms of the magnitude and the level of induction of IR-responsive miRNA species in hESCs. MiRNAome alterations are predicted to support the pluripotent state of irradiated hESCs, and mainly affect the cell cycle-, and alternative splicing-related processes. Thus, the fundamental role of the miRNAome in modulating the radiation response of hESCs is becoming increasingly appreciated by a scientific community, paving the way in identification of novel molecular targets of radiation in hESCs. The described mechanism elucidates the role of miRNAs in regulation of important molecular pathway governing the G(1)/S transition checkpoint before as well as after DNA damage.

## 7. Non-Targeted Effects of Ionizing Radiation Exposures in Human Embryonic Stem Cells

Until recently, a key paradigm that was dominating the radiation biology field for many decades held that the direct interaction of radiation, and/or radiation-induced free radicals, with unique cellular targets such as DNA molecules is a necessary and sufficient prerequisite for full realization of biological effects of IR exposures [3, 70]. More recently, however, a growing body of experimental evidence began to challenge this assumption by showing that IR could trigger secondary effects in nonirradiated cells [71–74]. These effects, termed radiation-induced bystander effects (RIBE), result from an intercellular communication between the irradiated cells and bystanders [72, 75]. It was shown that at least two mechanisms of intercellular communication are involved in RIBE, namely gap junction-mediated and secreted soluble factor-dependent signaling [76]. RIBE was found to occur in a number of experimental systems both *in vitro* and *in vivo* as a result of exposure to IR. RIBE may cause DNA damage and eventual death in these bystander cells. However, very little is known about RIBE in hESCs. Our study was the first to mechanistically interrogate RIBE in hESCs [77]. We irradiated hESCs (H9 line) with doses 0.2 Gy, 2 Gy, and 10 Gy of X-rays. Then using a medium-transfer protocol, we examined secreted soluble factor-dependent signaling between directly IR-exposed and bystander hESC cultures. The conditioned bystander medium transfer results showed no evidence for RIBE in hESCs by the criteria of induction of DNA damage and for apoptotic cell death as compared to nonirradiated cells [77]. These data indicate that hESCs might be less susceptible to damaging effects of RIBE signaling compared to fully differentiated adult human somatic cells [72, 77].

## 8. Future Perspectives

The data published in the literature regarding hESC responses to IR exposures, and summarized in this paper, show that many important aspects of such responses are still unknown, warranting additional efforts of researchers in the field. The results suggest that despite generally higher capacity of hESCs to repair DNA damage compared with differentiated human cells, pluripotent hESCs are prone to undergo massive apoptosis following relatively moderate doses of IR exposures. Many unknowns remain to be addressed and solved before a comprehensive understanding of hESC responses to IR begin to emerge. First, published data were obtained utilizing a rather limited repertoire of hESC lines out from dozens of available hESCs thus far [78, 79]. For example, only one hESC line was used in studies such as [16, 18, 26, 30, 50]; others used two hESC lines [17, 22, 32]; only rarely three [52, 69]. Almost all studies into biological effects of IR on hESCs were done with H1, H7, H9, and BG01V lines. Therefore, it is unclear if the findings obtained with these lines can be easily generalized into other much less researched hESC lines too. It seems imperative for scientists to go beyond those few hESC lines and explore other genetically distinct hESCs as well. Second, many hESC lines were shown to acquire genetic defects (copy-number changes, loss of heterozygosity, etc.) *in vitro* at high passage numbers [80–85], that could potentially influence their response to DNA damage and their checkpoint activation. Third, the effects of radiation were examined on bulk hESC populations, although the level of heterogeneity in hESC cultures might be quite high [86]. In addition, heterogeneity of cell cycle distribution within asynchronous hESC cultures may result in differences in a repertoire of gene expression amongst cells. This raises the necessity to apply novel methodological approaches to study IR responses in hESCs at a single-cell level. The importance of such research may stem from the fact that distinct hESC subpopulations may possess unique biological characteristics still not uncovered with traditional experimental techniques. Fourth, the radiosensitivity of human cells is routinely assessed with the clonogenic assay in radiobiology [87, 88]; however, there are numerous technical challenges in adoption of this assay to study the radioresponses of hESCs. These pluripotent cells grow in colonies, and attempts to obtain viable single-cell suspensions of hESCs required for clonogenic assay witnessed only very limited success, unless the inhibitors of specific pathways are used [89–91]. Hence, there is a need to develop novel techniques to examine hESC radiosensitivity beyond clonogenic assay. Fifth, to date, only limited data are available how hESCs respond to IR at a system biology level [16, 33, 49, 69]. Nothing is known about changes in proteome, metabolome, DNA methylome, histone modifications, and induction of genetic alterations in surviving hESCs genome-wide following IR exposures. It will be interesting to determine if radiation-induced genetic/epigenetic changes in hESCs, if any, confer the growth advantage to such cells using not only *in vitro*, but also *in vivo* models. This knowledge, especially integrated with results gathered using other “omics” high-throughput approaches, and put into the context of multiple hESC lines may ultimately provide researchers with a long-sought roadmap to address key fundamental issues in radiation biomedical science, and to pave the way for future more focused and detailed 

## References

[1] Thomson JA, Itskovitz-Eldor J, Shapiro SS (1998). Embryonic stem cell lines derived from human blastocysts. *Science*.

[2] Shamblott MJ, Axelman J, Wang S (1998). Derivation of pluripotent stem cells from cultured human primordial germ cells. *Proceedings of the National Academy of Sciences of the United States of America*.

[3] Iu BK (2001). Main principles of radiobiology. *Radiatsionnaia Biologiia, Radioecologiia*.

[4] Turesson I, Carlsson J, Brahme A (2003). Biological response to radiation therapy. *Acta Oncologica*.

[5] Preston RJ (2005). Radiation biology: concepts for radiation protection. *Health Physics*.

[6] De Santis M, Cesari E, Nobili E, Straface G, Cavaliere AF, Caruso A (2007). Radiation effects on development. *Birth Defects Research Part C*.

[7] McCollough CH, Schueler BA, Atwell TD (2007). Radiation exposure and pregnancy: when should we be concerned?. *Radiographics*.

[8] Fazel R, Krumholz HM, Wang Y (2009). Exposure to low-dose ionizing radiation from medical imaging procedures. *The New England Journal of Medicine*.

[9] Smith-Bindman R, Lipson J, Marcus R (2009). Radiation dose associated with common computed tomography examinations and the associated lifetime attributable risk of cancer. *Archives of Internal Medicine*.

[10] Daza P, Schübler H, McMillan TJ, Girod SC, Pfeiffer P (1997). Radiosensitivity and double-strand break rejoining in tumorigenic and non-tumorigenic human epithelial cell lines. *International Journal of Radiation Biology*.

[11] Rothkamm K, Löbrich M (2003). Evidence for a lack of DNA double-strand break repair in human cells exposed to very low X-ray doses. *Proceedings of the National Academy of Sciences of the United States of America*.

[12] Sokolov MV, Neumann RD, Panyutin IG (2007). Effects of DNA-targeted ionizing radiation produced by 5-[^125^I]iodo-2′-deoxyuridine on global gene expression in primary human cells. *BMC Genomics*.

[13] Sokolov MV, Smirnova NA, Camerini-Otero RD, Neumann RD, Panyutin IG (2006). Microarray analysis of differentially expressed genes after exposure of normal human fibroblasts to ionizing radiation from an external source and from DNA-incorporated iodine-125 radionuclide. *Gene*.

[14] Cabuy E, Newton C, Joksic G (2005). Accelerated telomere shortening and telomere abnormalities in radiosensitive cell lines. *Radiation Research*.

[15] Becher UM, Breitbach M, Sasse P (2009). Enrichment and terminal differentiation of striated muscle progenitors in vitro. *Experimental Cell Research*.

[16] Wilson KD, Sun N, Huang M (2010). Effects of ionizing radiation on self-renewal and pluripotency of human embryonic stem cells. *Cancer Research*.

[17] Momčilović O, Choi S, Varum S, Bakkenist C, Schatten G, Navara C (2009). Ionizing radiation induces ataxia telangiectasia mutated-dependent checkpoint signaling and G2 but not G1 cell cycle arrest in pluripotent human embryonic stem cells. *Stem Cells*.

[18] Filion TM, Qiao M, Ghule PN (2009). Survival responses of human embryonic stem cells to DNA damage. *Journal of Cellular Physiology*.

[19] Sokolov MV, Panyutin IV, Onyshchenko MI, Panyutin IG, Neumann RD (2010). Expression of pluripotency-associated genes in the surviving fraction of cultured human embryonic stem cells is not significantly affected by ionizing radiation. *Gene*.

[20] Qin H, Yu T, Qing T (2007). Regulation of apoptosis and differentiation by p53 in human embryonic stem cells. *Journal of Biological Chemistry*.

[21] Dumitru R, Gama V, Fagan BM (2012). Human embryonic stem cells have constitutively active bax at the golgi and are primed to undergo rapid apoptosis. *Molecular Cell*.

[22] Becker KA, Ghule PN, Therrien JA (2006). Self-renewal of human embryonic stem cells is supported by a shortened G1 cell cycle phase. *Journal of Cellular Physiology*.

[23] Becker KA, Stein JL, Lian JB, van Wijnen AJ, Stein GS (2007). Establishment of histone gene regulation and cell cycle checkpoint control in human embryonic stem cells. *Journal of Cellular Physiology*.

[24] Ghule PN, Becker KA, Harper JW (2007). Cell cycle dependent phosphorylation and subnuclear organization of the histone gene regulator p220NPAT in human embryonic stem cells. *Journal of Cellular Physiology*.

[25] Ghule PN, Dominski Z, Yang XC (2008). Staged assembly of histone gene expression machinery at subnuclear foci in the abbreviated cell cycle of human embryonic stem cells. *Proceedings of the National Academy of Sciences of the United States of America*.

[26] Becker KA, Ghule PN, Lian JB, Stein JL, van Wijnen AJ, Stein GS (2010). Cyclin D2 and the CDK substrate p220NPAT are required for self-renewal of human embryonic stem cells. *Journal of Cellular Physiology*.

[27] Kapinas K, Grandy R, Ghule P The abbreviated pluripotent cell cycle.

[28] Stein GS, Stein JL, van JWA (2012). The architectural organization of human stem cell cycle regulatory machinery. *Current Pharmaceutical Design*.

[29] Momčilović O, Navara C, Schatten G (2011). Cell cycle adaptations and maintenance of genomic integrity in embryonic stem cells and induced pluripotent stem cells. *Results and Problems in Cell Differentiation*.

[30] Becker KA, Stein JL, Lian JB, Van Wijnen AJ, Stein GS (2010). Human embryonic stem cells are pre-mitotically committed to self-renewal and acquire a lengthened G1 phase upon lineage programming. *Journal of Cellular Physiology*.

[31] Burma S, Chen BP, Murphy M, Kurimasa A, Chen DJ (2001). ATM Phosphorylates Histone H2AX in Response to DNA Double-strand Breaks. *Journal of Biological Chemistry*.

[32] Adams BR, Golding SE, Rao RR, Valerie K (2010). Dynamic dependence on ATR and ATM for double-Strand break repair in human embryonic stem cells and neural descendants. *PLoS ONE*.

[33] Sokolov MV, Panyutin IV, Panyutin IG, Neumann RD (2011). Dynamics of the transcriptome response of cultured human embryonic stem cells to ionizing radiation exposure. *Mutation Research*.

[34] Mantel C, Guo Y, Lee MR (2007). Checkpoint-apoptosis uncoupling in human and mouse embryonic stem cells: a source of karyotpic instability. *Blood*.

[35] Bárta T, Vinarský V, Holubcová Z (2010). Human embryonic stem cells are capable of executing G1/S checkpoint activation. *Stem Cells*.

[36] Neganova I, Vilella F, Atkinson SP (2011). An important role for CDK2 in G1 to S checkpoint activation and DNA damage response in human embryonic stem cells. *Stem Cells*.

[37] Maimets T, Neganova I, Armstrong L, Lako M (2008). Activation of p53 by nutlin leads to rapid differentiation of human embryonic stem cells. *Oncogene*.

[38] Svilar D, Goellner EM, Almeida KH, Sobol RW (2011). Base excision repair and lesion-dependent subpathways for repair of oxidative DNA damage. *Antioxidants and Redox Signaling*.

[39] Wilson DM, Kim D, Berquist BR, Sigurdson AJ (2011). Variation in base excision repair capacity. *Mutation Research*.

[40] Vermeulen W (2011). Dynamics of mammalian NER proteins. *DNA Repair*.

[41] Liu L, Lee J, Zhou P (2010). Navigating the nucleotide excision repair threshold. *Journal of Cellular Physiology*.

[42] Jiricny J (2006). The multifaceted mismatch-repair system. *Nature Reviews Molecular Cell Biology*.

[43] Kass EM, Jasin M (2010). Collaboration and competition between DNA double-strand break repair pathways. *FEBS Letters*.

[44] Holthausen JT, Wyman C, Kanaar R (2010). Regulation of DNA strand exchange in homologous recombination. *DNA Repair*.

[45] Mladenov E, Iliakis G (2011). Induction and repair of DNA double strand breaks: the increasing spectrum of non-homologous end joining pathways. *Mutation Research*.

[46] Lieber MR (2010). The mechanism of double-strand DNA break repair by the nonhomologous DNA end-joining pathway. *Annual Review of Biochemistry*.

[47] Moynahan ME, Jasin M (2010). Mitotic homologous recombination maintains genomic stability and suppresses tumorigenesis. *Nature Reviews Molecular Cell Biology*.

[48] Diderich K, Alanazi M, Hoeijmakers JHJ (2011). Premature aging and cancer in nucleotide excision repair-disorders. *DNA Repair*.

[49] Maynard S, Swistowska AM, Jae WL (2008). Human embryonic stem cells have enhanced repair of multiple forms of DNA damage. *Stem Cells*.

[50] Adams BR, Hawkins AJ, Povirk LF, Valerie K (2010). ATM-independent, high-fidelity nonhomologous end joining predominates in human embryonic stem cells. *Aging*.

[51] Fung H, Weinstock DM (2011). Repair at single targeted DNA double-strand breaks in pluripotent and differentiated human cells. *PLoS ONE*.

[52] Luo LZ, Gopalakrishna-Pillai S, Nay SL (2012). DNA repair in human pluripotent stem cells is distinct from that in non-pluripotent human cells. *PLoS ONE*.

[53] Rieger KE, Chu G (2004). Portrait of transcriptional responses to ultraviolet and ionizing radiation in human cells. *Nucleic Acids Research*.

[54] Sokolov M, Panyutin IG, Neumann R (2006). Genome-wide gene expression changes in normal human fibroblasts in response to low-LET gamma-radiation and high-LET-like 125IUdR exposures. *Radiation Protection Dosimetry*.

[55] Amundson SA, Do KT, Fornace AJ (1999). Induction of stress genes by low doses of gamma rays. *Radiation Research*.

[56] Besson A, Dowdy SF, Roberts JM (2008). CDK inhibitors: cell cycle regulators and beyond. *Developmental Cell*.

[57] Rødningen OK, Overgaard J, Alsner J, Hastie T, Børresen-Dale AL (2005). Microarray analysis of the transcriptional responseto single or multiple doses of ionizing radiation in human subcutaneous fibroblasts. *Radiotherapy and Oncology*.

[58] Calvanese V, Fraga MF (2012). Epigenetics of embryonic stem cells. *Advances in Experimental Medicine and Biology*.

[59] Mattout A, Meshorer E (2010). Chromatin plasticity and genome organization in pluripotent embryonic stem cells. *Current Opinion in Cell Biology*.

[60] Meissner A (2010). Epigenetic modifications in pluripotent and differentiated cells. *Nature Biotechnology*.

[61] Bibikova M, Chudin E, Wu B (2006). Human embryonic stem cells have a unique epigenetic signature. *Genome Research*.

[62] Altun G, Loring JF, Laurent LC (2010). DNA methylation in embryonic stem cells. *Journal of Cellular Biochemistry*.

[63] Bock C, Kiskinis E, Verstappen G (2011). Reference maps of human es and ips cell variation enable high-throughput characterization of pluripotent cell lines. *Cell*.

[64] Chen PY, Feng S, Joo JW, Jacobsen SE, Pellegrini M (2011). A comparative analysis of DNA methylation across human embryonic stem cell lines. *Genome Biology*.

[65] Laurent LC, Chen J, Ulitsky I (2008). Comprehensive microRNA profiling reveals a unique human embryonic stem cell signature dominated by a single seed sequence. *Stem Cells*.

[66] Aranda P, Agirre X, Ballestar E (2009). Epigenetic signatures associated with different levels of differentiation potential in human stem cells. *PLoS ONE*.

[67] Dasa D, Marek M, Tomas B (2012). MicroRNAs regulate p21(Waf1/cip1) protein expression and the DNA damage response in human embryonic stem cells. *Stem Cells*.

[68] Qi J, Yu JY, Shcherbata HR (2009). microRNAs regulate human embryonic stem cell division. *Cell Cycle*.

[69] Sokolov MV, Panyutin IV, Neumann RD (2012). Unraveling the global microRNAome responses to ionizing radiation in human embryonic stem cells. *PLoS ONE*.

[70] Elkind MM, Whitmore GF (1967). *The Radiobiology of Cultured Mammalian Cells*.

[71] Nagasawa H, Little JB (1992). Induction of sister chromatid exchanges by extremely low doses of alpha-particles. *Cancer Research*.

[72] Sokolov MV, Smilenov LB, Hall EJ, Panyutin IG, Bonner WM, Sedelnikova OA (2005). Ionizing radiation induces DNA double-strand breaks in bystander primary human fibroblasts. *Oncogene*.

[73] Sokolov MV, Dickey JS, Bonner WM, Sedelnikova OA (2007). *γ*-H2AX in bystander cells: not just a radiation-triggered event, a cellular response to stress mediated by intercellular communication. *Cell Cycle*.

[74] Dickey JS, Baird BJ, Redon CE, Sokolov MV, Sedelnikova OA, Bonner WM (2009). Intercellular communication of cellular stress monitored by *γ*-H2AX induction. *Carcinogenesis*.

[75] Mothersill C, Seymour C (1997). Medium from irradiated human epithelial cells but not human fibroblasts reduces the clonogenic survival of unirradiated cells. *International Journal of Radiation Biology*.

[76] Hamada N, Matsumoto H, Hara T, Kobayashi Y (2007). Intercellular and intracellular signaling pathways mediating ionizing radiation-induced bystander effects. *Journal of Radiation Research*.

[77] Sokolov MV, Neumann RD (2010). Radiation-induced bystander effects in cultured human stem cells. *PLoS ONE*.

[78] Adewumi O, Aflatoonian B, Ahrlund-Richter L (2007). Characterization of human embryonic stem cell lines by the International Stem Cell Initiative. *Nature Biotechnology*.

[79] Allegrucci C, Young LE (2007). Differences between human embryonic stem cell lines. *Human Reproduction Update*.

[80] Närvä E, Autio R, Rahkonen N (2010). High-resolution DNA analysis of human embryonic stem cell lines reveals culture-induced copy number changes and loss of heterozygosity. *Nature Biotechnology*.

[81] Werbowetski-Ogilvie TE, Bossé M, Stewart M (2009). Characterization of human embryonic stem cells with features of neoplastic progression. *Nature Biotechnology*.

[82] Spits C, Mateizel I, Geens M (2008). Recurrent chromosomal abnormalities in human embryonic stem cells. *Nature Biotechnology*.

[83] Wu H, Kim KJ, Mehta K (2008). Copy number variant analysis of human embryonic stem cells. *Stem Cells*.

[84] Amps K, Andrews PW, Anyfantis G (2011). Screening ethnically diverse human embryonic stem cells identifies a chromosome 20 minimal amplicon conferring growth advantage. *Nature Biotechnology*.

[85] Baker DEC, Harrison NJ, Maltby E (2007). Adaptation to culture of human embryonic stem cells and oncogenesis *in vivo*. *Nature Biotechnology*.

[86] King FW, Ritner C, Liszewski W (2009). Subpopulations of human embryonic stem cells with distinct tissue-specific fates can be selected from pluripotent cultures. *Stem Cells and Development*.

[87] Fertil B, Malaise EP (1981). Inherent cellular radiosensitivity as a basic concept for human tumor radiotherapy. *International Journal of Radiation Oncology Biology Physics*.

[88] Williams JR, Zhang Y, Zhou H (2008). Overview of radiosensitivity of human tumor cells to low-dose-rate irradiation. *International Journal of Radiation Oncology Biology Physics*.

[89] Watanabe K, Ueno M, Kamiya D (2007). A ROCK inhibitor permits survival of dissociated human embryonic stem cells. *Nature Biotechnology*.

[90] Barbaric I, Jones M, Buchner K, Baker D, Andrews PW, Moore HD (2011). Pinacidil enhances survival of cryopreserved human embryonic stem cells. *Cryobiology*.

[91] Ohgushi M, Matsumura M, Eiraku M (2010). Molecular pathway and cell state responsible for dissociation-induced apoptosis in human pluripotent stem cells. *Cell Stem Cell*.

